# Age- and Experience-Related Plasticity of ATP-Mediated Signaling in the Neocortex

**DOI:** 10.3389/fncel.2019.00242

**Published:** 2019-05-29

**Authors:** Ulyana Lalo, Alexander Bogdanov, Yuriy Pankratov

**Affiliations:** ^1^School of Life Sciences, Gibbet Hill Campus, University of Warwick, Coventry, United Kingdom; ^2^Institute for Chemistry and Biology, Immanuel Kant Baltic Federal University, Kaliningrad, Russia

**Keywords:** ageing, synaptic strength, glia-neuron interaction, diet, exercise, GABA receptor A, AMPA receptor, calcium

## Abstract

There is growing recognition of the important role of interaction between neurons and glial cells for brain longevity. The extracellular ATP have been shown to bring significant contribution into bi-directional glia-neuron communications, in particular into astrocyte-driven modulation of synaptic plasticity. To elucidate a putative impact of brain aging on neuron-glia networks, we explored the aging-related plasticity of the purinoreceptors-mediated signaling in cortical neurons and astrocytes. We investigated the age- and experience-related alterations in purinergic components of neuronal synaptic currents and astroglial calcium signaling in the layer2/3 of neocortex of mice exposed to the mild caloric restriction (CR) and environmental enrichment (EE) which included *ad libitum* physical exercise. We observed the considerable age-related decline in the neuronal P2X receptor-mediated miniature spontaneous currents which originated from the release of ATP from both synapses and astrocytes. We also found out that purinergic astrocytic Ca^2+^-signaling underwent the substantial age-related decline but EE and CR rescued astroglial signaling, in particular mediated by P2X1, P2X1/5, and P2Y1 receptors. Our data showed that age-related attenuation in the astroglial calcium signaling caused a substantial decrease in the exocytosis of ATP leading to impairment of astroglia-derived purinergic modulation of excitatory synaptic currents and GABAergic tonic inhibitory currents. On a contrary, exposure to EE and CR, which enhanced purinergic astrocytic calcium signaling, up-regulated the excitatory and down-regulated the inhibitory currents in neurons of old mice, thus counterbalancing the impact of aging on synaptic signaling. Combined, our results strongly support the physiological importance of ATP-mediated signaling for glia-neuron interactions and brain function. Our data also show that P2 purinoreceptor-mediated communication between astrocytes and neurons in the neocortex undergoes remodeling during brain aging and decrease in the ATP release may contribute to the age-related impairment of synaptic transmission.

## Introduction

Adaptation of mammalian brain to environmental and biochemical challenges across a life-time is associated with remodeling of synaptic contacts and plastic changes in the neural networks (Hillman et al., 2008; Nithianantharajah and Hannan, 2009; van Praag, 2009; Mercken et al., 2012; Merzenich et al., 2014). The responsiveness of neural networks and synapses to enriched environment (EE), physical activity, and caloric restriction (CR) (Hillman et al., 2008; Nithianantharajah and Hannan, 2009; van Praag, 2009; Mercken et al., 2012; Merzenich et al., 2014) provides an opportunity to ameliorate the negative consequences of aging on cognitive function. Still, cellular and molecular mechanisms underlying effects of exercise and CR on synaptic plasticity are yet to be fully understood.

It is now widely acknowledged that maintenance and modification of synaptic networks depends on the interaction between neurons and neuroglia (Araque et al., 2014; Hulme et al., 2014; Gundersen et al., 2015; De Strooper and Karran, 2016). However, a great deal of information on this interaction has been derived from the experiments on young animals, whereas the age-dependent modification of glia-neuron communications remains unknown.

There is an accumulating evidence of importance of extracellular ATP for transmitting signals between brain neurons and glia. ATP can be released from nerve terminals, mainly by exocytosis, and astrocytes, both by vesicular release and diffusion through the plasmalemmal channels (Fields and Stevens, 2000; Pangrsic et al., 2007; Abbracchio et al., 2009; Butt, 2011; Khakh and North, 2012; Lalo et al., 2014a). The role for synaptically released ATP in neuronal signaling has been established for several brain areas, such as medial habenula, hippocampus and somatosensory cortex (Robertson et al., 1999; Pankratov et al., 2003, 2007, 2009). Release of ATP from astrocytes represents a powerful pathway of glia-neuron interaction implicated in the synaptic plasticity (Araque et al., 2014; Rasooli-Nejad et al., 2014; Pankratov and Lalo, 2015; Lalo et al., 2016), meta-plasticity (Hulme et al., 2014; Lalo et al., 2018), and neurological disorders (Butt, 2011; Rodrigues et al., 2015; Rivera et al., 2016; Verkhratsky et al., 2017). Apart from mediating a significant component of glia-to-neuron signaling, astrocyte-derived ATP can also act in autocrine manner activating purinoreceptor-mediated Ca^2+^-signaling in astroglial networks (Fields and Stevens, 2000; Fields and Burnstock, 2006; Gourine et al., 2010; Butt, 2011; Lalo et al., 2011b; Wells et al., 2015).

Effects of ATP as neuro- and gliotransmitter are mediated by ionotropic P2X and metabotropic P2Y purinoreceptors which are abundantly expressed in the brain and can bring significant contribution to the intracellular Ca^2+^-signaling in various types of cells and (Burnstock, 2007; Abbracchio et al., 2009; Khakh and North, 2012; Pankratov and Lalo, 2014). Acting via P2X and P2Y receptors, extracellular ATP can exert various neuro-modulatory effects on excitatory and inhibitory synaptic transmission both at pre- and post-synaptic loci (Khakh and North, 2012; Boue-Grabot and Pankratov, 2017).

Hence, ATP as extracellular transmitter can bring significant contribution into the bi-directional communications between neurons and astrocytes. Our recent data highlighted an important role of glia-neuron interactions in the experience-induced metaplasticity in aging brain (Lalo et al., 2018). We also reported previously that ATP-mediated signaling in astrocytes can undergo significant age-dependent remodeling. Our data, as well as reports of other groups, suggest that alterations in neuronal and glial ATP-mediated signaling can be involved in (patho)physiological changes in synaptic transmission in aging and neurodegenerative diseases (Delekate et al., 2014; Verkhratsky et al., 2017; Zorec et al., 2017; Lalo et al., 2018). Still, aging-related plasticity of purinergic component of glia-neuron communications remains largely unexplored. Here we report in depth analysis of the age-dependent changes in purinergic signaling in neocortical astrocytes and neurons which we performed aiming to understand how brain aging affects signaling in tripartite synapse. We also elucidated the putative impact of environmental enrichment (EE) and CR on purinergic signaling.

## Materials and Methods

All animal work has been carried out in accordance with United Kingdom legislation and “3R” strategy; research has not involved non-human primates. Experiments were performed in astrocytes and pyramidal neurons of somatosensory cortex of transgenic mice expressing enhanced green fluorescent protein (EGFP) under control of the human glial fibrillary acidic protein (GFAP) promoter (GFAP-EGFP mice; see Lalo et al., 2008). We used mice of four age groups: 3–6 (average 4.2 ± 1.1) weeks (young), 3–6 (4.4 ± 1.2) months (adult), 8–12 (10.3 ± 1.4) months (old), and 16–28 (21.4 ± 3.2) months (very old). We compared animals kept under standard housing conditions (SH) vs animals exposed to the enriched environment (EE) from birth (Correa et al., 2012), including *ad libitum* access to the running wheel, or kept on mild CR diet (food intake individually regulated to maintain the body weight loss of 10–15%) for 4–6 weeks.

### Slice and Cell Preparation

Mice were anesthetized by halothane and then decapitated, in accordance with United Kingdom legislation. Brains were removed rapidly after decapitation and placed into ice-cold physiological saline containing (mM): NaCl 130, KCl 3, CaCl_2_ 0.5, MgCl_2_ 2.5, NaH_2_PO_4_ 1, NaHCO_3_ 25, glucose 15, and pH of 7.4 gassed with 95% O_2_ – 5% CO_2_. Transverse slices (260 μm) were cut at 4°C and then placed in physiological saline containing (mM): NaCl 130, KCl 3, CaCl_2_ 2.5, MgCl_2_ 1, NaH_2_PO_4_ 1, NaHCO_3_ 22, glucose 15, pH of 7.4 gassed with 95% O_2_ - 5% CO_2_, and kept for 1.5 – 5 h prior to cell isolation and recording.

Astrocytes were initially identified by their morphology under DIC observation and EGFP fluorescence. After the recordings, the identification of astrocyte was confirmed via functional properties (high potassium conductance, low input resistance, and strong activity of glutamate transporters) as described previously (Lalo et al., 2014a; Rasooli-Nejad et al., 2014; Pankratov and Lalo, 2015). To facilitate the high-quality whole-cell recordings in the brain tissue slices of old mice, tissue slices were treated with vibrating glass ball remove the upper layer of dead cells and expose healthy neurons (Lalo and Pankratov, 2017).

Whole-cell voltage clamp recordings from cortical neurones and astrocytes cells were made with patch pipettes (4 – 5 MΩ) filled with intracellular solution (in mM): 60 CsCl, 50 CsGluconate, 10 NaCl, 10 HEPES, 5 MgATP, 1 D-Serine, 0.1 EGTA, and pH 7.35; Currents were monitored using an MultiClamp 700B patch-clamp amplifier (Axon Instruments, United States) filtered at 2 kHz and digitized at 4 kHz. Experiments were controlled by Digidata 1440A data acquisition board (Axon Instruments, United States) and WinWCP software (University of Strathclyde, United Kingdom); data were analyzed by self-designed software. Liquid junction potentials were compensated with the patch-clamp amplifier. The series and input resistances were, respectively 5–7 MΩ and 600–1100 MΩ; both series and input resistance varied by less than 20% in the cells accepted for analysis. In the younger mice (up to age of 6 months), the 90% of neurons tested showed acceptable parameters (as above) of whole-cell recordings, in the older mice, only 60% of neurons tests were suitable for long-time high-quality whole-cell recordings.

For activation of synaptic responses, axons originating from layer IV–VI neurons were stimulated with a bipolar coaxial electrode (WPI, United States) placed in layer V close to the layer IV border, approximately opposite the site of recording; stimulus duration was 300 μs, train of 5 stimuli was delivered at 100 Hz. The stimulus magnitude was set 3 – 4 times higher than the minimal stimulus necessary to elicit a response in layer II pyramidal neurons (Rasooli-Nejad et al., 2014; Pankratov and Lalo, 2015; Lalo et al., 2016).

### Multi-Photon Fluorescent Ca^2+^-Imaging in Astrocytes

To monitor the cytoplasmic free Ca^2+^concentration ([Ca^2+^]_i_) *in situ*, astrocytes of neocortical slices were loaded via 30 min incubation with 1 μM of Rhod-2AM (dnSNARE mice) or Oregon Green Bapta-2AM and sulforhodamine 101 (wild-type and MSK1 KD mice) at 33°C. Two-photon images of neurons and astrocytes were acquired at 5 Hz frame-rate using a Zeiss LSM 7MP multi-photon microscope coupled to a Spectra-Physics MaiTai pulsing laser; experiments were controlled by ZEN LSM software (Carl Zeiss, Germany). Images were further analyzed off-line using ZEN LSM (Carl Zeiss) and ImageJ (NIH) software (Schneider et al., 2012). The [Ca^2+^]_i_ levels were expressed as ΔF/F ratio averaged over a region of interest (ROI). For analysis of spontaneous Ca^2+^–transients in astrocytes, 5–10 ROIs located over the astrocytic branches and 1 ROI located over the soma were chosen. Overall Ca^2+^ -response to receptors agonists or synaptic stimulation was quantified using an ROI covering the whole cell image.

### Data Analysis

All data are presented as mean ± SD and the statistical significance of differences between data groups was tested by two-tailed unpaired *t*-test, unless indicated otherwise. For all cases of statistical significance reported, the statistical power of the test was 0.8–0.9.

The spontaneous transmembrane currents recorded in neurons were analyzed off-line using methods described previously (Lalo et al., 2014a, 2016). The amplitude distributions of spontaneous and evoked currents were analyzed with the aid of probability density functions and likelihood maximization techniques; all histograms shown were calculated as probability density functions. The amplitude distributions were fitted with either multi-quantal binomial model or bi-modal function consisting of two Gaussians with variable peak location, width and amplitude. Parameters of models were fit using likelihood maximization routine.

## Results

### Age- and Environment-Related Alterations in Astroglial Ca^2+^-Signaling

**FIGURE 1:**
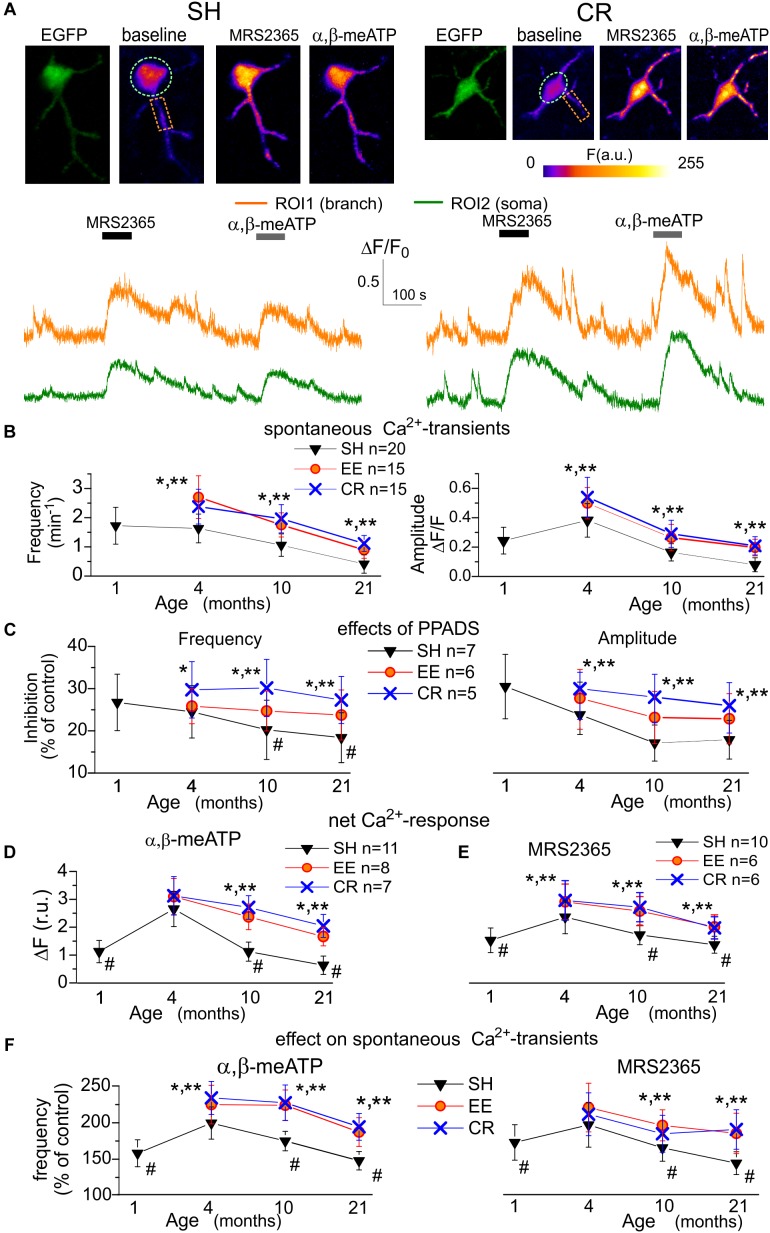
Age- and experience-related changes in the purinergic astrocytic Ca^2+^ signaling. **(A)** Representative multi-photon images of EGFP fluorescence and pseudo-color images of Rhod-2 fluorescence recorded in the astrocytes of old GFAP-EGFP (GFEC) mice kept in the standard housing conditions (SH) and exposed to calorie restriction diet (CR). Images were acquired before (baseline) and after the application of specific agonists of P2X (α,β-me ATP) and P2Y1 (MRS2365) receptors. Graphs below show the time course of Rhod-2 fluorescence averaged over regions of interests (ROI) indicated in the fluorescence images. Note the marked increase in the spontaneous Ca^2+^-elevations and Ca^2+^-responses to α,β-me ATP and MRS2365. **(B)** The pooled data on the peak amplitude and frequency of baseline spontaneous Ca^2+^-transients recorded in the astrocytes of mice of different age and treatment groups. **(C)** The pooled data on the effect of P2 purinoreceptor antagonist PPADS on the peak amplitude and frequency of spontaneous Ca^2+^-transients. **(D,E)** The pooled data on the net responses to application of α,β-me ATP and MRS2365 in the different age groups. Net response was evaluated as an integral Ca^2+^-signal measured within 3 min after stimulation and normalized to the baseline integral Ca^2+^ signal. **(F)** The pooled data on the changes in the frequency of spontaneous Ca^2+^-transients (relative to the baseline) registered in the neocortical astrocytes within 5 min after application of α,β-me ATP and MRS2365; the cell numbers are the same as in **(D,E)**. Data in the panels **(B–F)** are shown as mean ± SD for the 6–12 astrocytes (as indicated) from 3 to 6 animals. The hush symbols (#) indicate statistical significance (*P* < 0.02) of the difference between the adult SH mice and SH mice of other age groups. Asterisks (^∗^, ^∗∗^) correspondingly indicate statistical significance (*P* < 0.05) of the effect of EE- or CR-treatment (as compared to SH mice of the same age group). Note the significant increase in the spontaneous and agonist-evoked Ca^2+^-signaling in astrocytes of mice exposed to EE and CR.

We recorded spontaneous and evoked cytosolic Ca^2+^-transients in the branches and somata of neocortical astrocytes of EFGP/ GFAP mice of four age groups: young (average 4.2 weeks), adult (4.4 months), old (10.3 months), and very old (21.4 months). Mice were kept under SH or exposed either to enriched environment (EE) or CR; the details are given in *Methods*. The astrocytes in brain slices were loaded with fluorescent Ca^2+^-probe Rhod-2AM and identified by their green fluorescence (Figure 1A) and, after the recordings, by their characteristic electrophysiological signature (Lalo et al., 2011a, 2014b).

In baseline, before synaptic stimulation or agonists application, astrocytes of mice of all age groups exhibited spontaneous Ca^2+^-transients, which were more prominent in the astrocytic branches (Figure 1A). The average frequency of spontaneous Ca^2+^-transients in astrocytes of young SH mice was 0.23 ± 0.11 min^-1^ in the soma but reached of 1.38 ± 0.41 min^-1^ in the astrocytic arborization (i.e., calculated for the whole cell image except soma). For the sake of simplicity, the further analysis of alterations in the spontaneous Ca^2+^-signaling used data evaluated for the whole astrocyte image. This predominantly reflected a situation in the astrocytic branches but would not distort the overall picture since the changes in purinergic signaling in the somata followed the same trend as in branches. Also, it is widely recognized that Ca^2+^-events relevant for astroglia functions and glia-neuron interaction occur mainly in the astrocytic processes (Khakh and McCarthy, 2015; Bazargani and Attwell, 2016; Agarwal et al., 2017).

We observed a moderate increase in the amplitude and frequency of spontaneous astrocytic Ca^2+^-transients of SH mice during maturation (i.e., between age groups I and II) which was the followed by strong age-dependent decrease (Figure 1B). Exposure to the EE or CR significantly increased the spontaneous astroglial Ca^2+^-signaling in the old mice (Figure 1B). To evaluate the overall contribution of ATP receptors to spontaneous astroglial signaling, we applied purinoreceptor antagonists PPADS which, in concentration used (10 μM), efficiently and selectively inhibits P2X and P2Y receptors. The application of PPADS partially inhibited spontaneous Ca^2+^ events in the astrocytes of mice of all ages and treatments. The relative effect of PPADS on the amplitude and frequency of Ca^2+^ -events was correspondingly 30.5 ± 7.8% and 26.7 ± 6.7% in the astrocytes of young SH mice with a tendency of moderate decrease in the old age (Figure 1C). Interestingly, exposure to CR led to the notable increase in the relative contribution of P2 purinoreceptors to astroglial Ca^2+^ -transients (Figure 1C). Hence, the age- and experience-related alterations in the spontaneous Ca^2+^ -transients might be attributed to the changes in the functional expression of P2X and/or P2Y receptors as important components of astrocytic signaling.

As previous works suggested P2X_1_ and P2Y_1_ subunits-containing receptors to bring main contribution to purinergic signaling in cortical astrocytes (Lalo et al., 2008; Butt, 2011; Di Castro et al., 2011; Mederos et al., 2019) we applied their agonists α,β-me ATP (10 μM) and MRS2365 (10 nM) to activate correspondingly ionotropic and metabotropic components of Ca^2+^-signaling (Figure 1A,D,E). Application of both agonists induced notable Ca^2+^-responses in the neocortical astrocytes of mice of all age groups (Figure 1A,D,E). Also, both α,β-me ATP and MRS2365 significantly increased the frequency of spontaneous Ca^2+^-transients (Figure 1F). The P2X-mediated response and effect of α,β-meATP on spontaneous signaling showed significant age-related decline but were rescued in the astrocytes of EE and CR-exposed mice (Figure 1D,F). In contrast to the P2X-mediated component, the metabotropic MRS2365-evoked Ca^2+^-response showed a moderate age-dependent decline (Figure 1E). Still, effects of EE and CR on P2Y_1_-mediated astrocytic signaling in the old mice were statistically significant.

**FIGURE 2:**
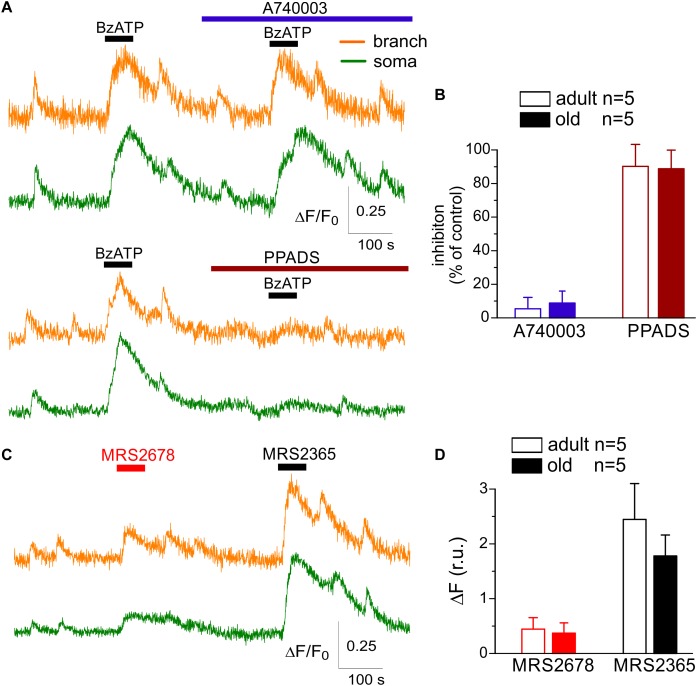
Action of subtype-specific drugs on Ca^2+^-signaling in cortical astrocytes. **(A,B)** Effect of P2X7 subtype-specific drugs. **(A)** Representative traces of Rhod-2 fluorescence recorded in the branches and somata of astrocytes of old SH mice. Fluorescence was measured before and after the application of P2X7 receptor agonist BzATP in control and in the presence of highly selective P2X7 antagonist A740003 and general P2 purinoreceptor antagonist PPADS. **(B)** The pooled data on the relative inhibitory effects of A740003 and PPADS on the net Ca^2+^-response to BzATP recorded in astrocytes of adult (4 month-old) and old (10 month-old) SH mice. The net response was evaluated as an integral Ca^2+^-signal measured within 3 min after stimulation, averaged over the whole cell image and normalized to the baseline integral Ca^2+^ signal. Note the lack of effect of P2X7-selective inhibitor A740003. **(C,D)** Effect of P2Y2 subtype-specific agonist MRS2678. **(D)** Representative traces of Rhod-2 fluorescence recorded in the branches and somata of astrocytes of old SH mice before and after application of selective agonists P2Y2 (MRS2678) and P2Y1 receptors (MRS2365). **(D)** The pooled data on the net Ca^2+^-response to MRS2365 and MRS2678 recorded in astrocytes of adult (4 month-old) and old (10 month-old) SH mice.

Since age-related alterations in the repertoire of P2 purinoreceptors expressed in astrocytes could not be ruled out *a priori*, we also tested the effects of drugs selective for P2X_7_, P2X_4_, and P2Y_2_ receptors (Figure 2). Although we observed a notable elevation of cytosolic Ca^2+^ in astrocytes after application of P2X7 receptor agonist BzATP (100 μM), these Ca^2+^-responses were weakly sensitive to the specific P2X7 antagonist A740003 (1 μM) but were effectively inhibited by PPADS in moderate (10 μM) concentrations (Figure 2A,B). In parallel, application of P2X7 antagonist A740003 did not had a significant inhibitory effect of the spontaneous Ca^2+^-transients in astrocytes. These results argue against a significant contribution of P2X_7_ receptors in the purinergic signaling in neocortical astrocytes, at least in our experimental paradigm.

We also observed only marginal inhibitory effects of selective P2X_4_ antagonist 5-BDBD on the amplitude and frequency of spontaneous astrocytic Ca^2+^-transients in the SH mice of all age groups (overall 3.8 ± 3.1%, *n* = 14; data not shown). In contrast, application of P2Y_2_ subtype-specific agonist MRS2678 (10 μM) elicited a modest elevation of cytosolic Ca^2+^ in the branches and soma of astrocytes in all age groups (Figure 2C,D); these Ca^2+^-responses were not sensitive to the specific P2Y_1_ antagonist. The P2Y_2_-mediated Ca^2+^-transients reached about 10–15% of signals activated via P2Y_1_ receptors and exhibited a weak age-dependent decline (Figure 2C).

These results strongly support the dominant role of P2X_1_ and P2Y_1_ subunit-containing receptors in the purinergic signaling in neocortical astrocytes and suggest that surface expression of functional purinoreceptors can undergo significant remodeling across a life-time and in response to the experience and diet. Age-related changes in the surface expression of P2 receptors can alter the responsiveness of astrocytes to the ATP released from the neighboring synapses as a co-transmitter (Pankratov et al., 2007; Lalo et al., 2016) and, thereby, affect their ability to monitor neuronal activity. To explore this, we investigated the changes in the Ca^2+^-responses of astrocytes to the stimulation of surrounding synapses.

### Synaptically Activated Ca^2+^-Transients in Cortical Astrocytes

**FIGURE 3:**
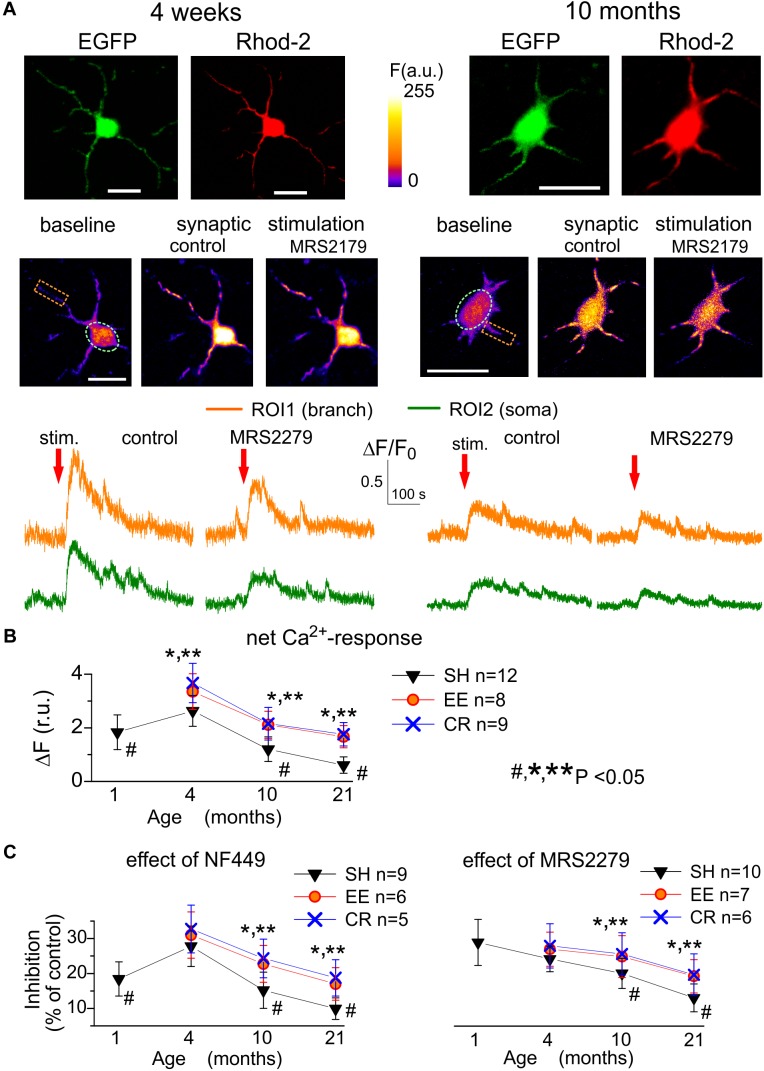
Age- and experience-related plasticity of astroglial Ca^2+^-response to synaptic stimulation. Ca^2+^ -signaling was monitored in cortical astrocytes loaded with Rhod-2AM using multi-photon fluorescent microscopy similar to the Figure 1. **(A)** Representative multi-photon images of EGFP fluorescence and pseudo-color images of Rhod-2 fluorescence recorded in the astrocytes of young (4 weeks) and very old (11 months) SH mice before (baseline) and after the stimulation (stim.) of cortical afferents under control conditions and in the presence of selective P2Y1 antagonist MRS2279 (10 nM). **(B,C)** The pooled data on the net responses of astrocytes (quantified similar to Figure 1) to the synaptic stimulation **(B)** and inhibitory effects of selective antagonists of P2X1 and P2Y1 receptors **(C)**. Data in the panels **(B,C)** are shown as mean ± SD for the 5–12 astrocytes (as indicated) from 3 to 5 animals. The hush symbols (#) indicate statistical significance (*P* < 0.02) of the difference between the adult SH mice and SH mice of other age groups. Asterisks (^∗^, ^∗∗^) correspondingly indicate statistical significance (*P* < 0.05) of the effect of EE- or CR-treatment (as compared to SH mice of the same age group). Note the significant increase in the synaptically evoked Ca^2+^-signaling in astrocytes of mice exposed to EE and CR.

As in our previous study of electrical signaling in astrocytes (Lalo et al., 2011a), we recorded synaptically activated Ca^2+^-responses evoked in the layer II/III astrocytes by the stimulation of neuronal afferents originating from layers IV-VI (Figure 3A). The synaptically evoked astroglial Ca^2+^-signaling moderately increased in the mature adult animals and then exhibited the significant age-dependent decline both in the somata and branches (Figure 3A,B). An exposure to EE and CR caused statistically significant increase in the evoked astrocytic Ca^2+^-response in mice of all age groups (Figure 3B).

As several different types of receptors, including NMDA, mGluR, and CB1, could participate in Ca^2+^-responses of astrocytes to synaptic stimulation (Lalo et al., 2008; Palygin et al., 2010; Rasooli-Nejad et al., 2014), we dissected the contribution of P2X and P2Y receptors pharmacologically, by applying a range of specific antagonists. We used highly subtype-specific antagonists NF449 (10 nM) and MRS2279 (300 nM) targeting correspondingly P2X_1_/P2X_1-5_ and P2Y_1_ receptors which were expected to bring major contribution into astroglial purinergic signaling in young mice (Lalo et al., 2008, 2011a; Butt, 2011).

The inhibitory effect of NF449 on the synaptically activated Ca^2+^-transients reached 18.7 ± 4.9% (*n* = 9, *P* < 0.05) in the young SH mice (Figure 3C) and showed moderate increase in the adult mice but declined sharply in the old and very old age. The age-related decline in the ionotropic purinergic (NF449-sensitive) Ca^2+^-signaling was partially ameliorated in the astrocytes of EE and CR-exposed mice (Figure 3C). In contrast to the P2X-mediated component, the metabotropic MRS2279-sensitive component of synaptically evoked Ca^2+^-transients showed a sharp decrease in old and very old age and rather modest EE- and CR-induced enhancement (Figure 3D).

Interestingly, both P2X- and P2Y-mediated components of synaptically activated response (Figure 3) showed steeper age-related decline and were less susceptible to EE and CR as their agonist-induced counterparts (Figure 1). The most likely explanation could be that, in parallel to alterations in the density of purinoreceptors, the release of ATP from synaptic terminals also underwent considerable changes. The decline in the synaptic release of ATP should have led to alterations in the purinergic synaptic currents in neurons which we explored in the next series of experiments.

### Age- and Environment-Related Alterations in Neuronal Purinergic Signaling

**FIGURE 4:**
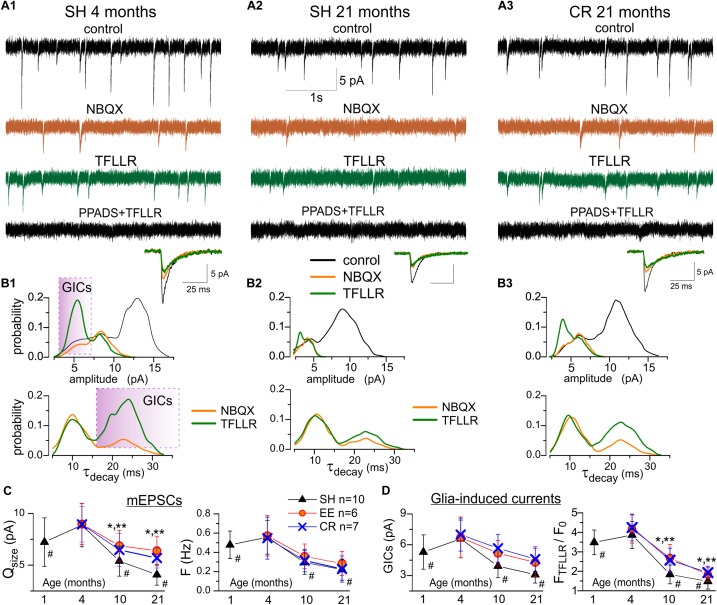
Age- and experience-related alterations in purinergic currents in neocortical neurons. The spontaneous transmembrane currents were recorded in the layer 2/3 pyramidal neurons of neocortical slices of the adult **(A1,B1)** and very old mice **(A2,3,B2,3)** at holding potential of –80 mV in presence of picrotoxin (100 μM). **(A1–3)**, *upper row* shows the representative transmembrane currents recorded before the application of NBQX (control), the AMPA receptors-mediated mEPSCs comprise the main fraction of spontaneous events; two *middle rows* show the non-glutamatergic inward spontaneous currents, recoded under NBQX before and 1 min after activation of Ca^2+^ -signaling in the astrocytes by selective agonist of astroglial PAR-1 receptors (TFLLR 10 μM); *bottom row* show transmembrane currents recorded after inhibition of P2X receptors with selective antagonists PPADS and 5-BDBD. The inserts at the bottom show an average mEPSCs waveforms under different conditions. Note the large increase in the number of non-glutamatergic currents in the adult mice under TFLLR and their disappearance under PPADS and 5-BDBD. **(B1–3)** the corresponding distributions (probability density functions) of the amplitude (*upper row*), and decay time (*lower row*) of spontaneous currents recorded before and after application of NBQX and TFLLR. Distributions reveal the presence of distinct population of spontaneous currents of smaller amplitude and slower kinetics in mice of both age groups. Stimulation of astrocytes with TFLLR significantly increases the peaks corresponding to smaller and slower sEPSCs, thus verifying their origin from astroglial release of ATP, as previously reported in Lalo et al. (2014a). Shaded areas indicate the range of decay times and amplitudes used to discriminate the glia-induced currents (GICs) from the currents of synaptic origin (mEPSCs) for the subsequent analysis of age-related plasticity **(C,D)**. **(C)** Pooled data on the quantal size (determined for each neuron as position of corresponding peak at the amplitude histogram) and frequency of purinergic mEPSCs in the mice of different age and experience groups. **(D)** pooled data on the quantal size of relative TFLLR-induced increase in the frequency of purinergic GICs. The data in the panels **(C,D)** are shown as mean ± SD for the 6–10 neurons (as indicated in **C**) from 3 to 4 animals. The hush symbols (#) indicate statistical significance (*P* < 0.02) of the difference between the adult SH mice and SH mice of other age groups. Asterisks (^∗^, ^∗∗^) correspondingly indicate statistical significance (*P* < 0.05) of the effect of EE- or CR-treatment (as compared to SH mice of the same age group). Note the significant decrease in the quantal size and frequency of purinergic currents in the old and very old mice (10–21 months) and increase in the synaptic and glia-induced neuronal purinergic signaling in mice exposed to EE and CR.

We have shown in the series of previous work that cortical pyramidal neurons of young mice express ionotropic P2X purinoreceptors which can be activated by release of ATP from both synaptic terminals and astrocytes (Lalo et al., 2014a, 2016). So, it might be plausible to detect P2X-mediated spontaneous currents in neurons of older animals. We recorded the whole-cell currents in neocortical pyramidal neurons at a membrane potential of -80 mV in the presence of picrotoxin (100 μM), D-APV (30 μM), and NBQX (30 μM), to eliminate the signals mediated correspondingly by the GABAA, NMDA, and AMPA receptors (Figure 4). We observed the residual non-glutamatergic miniature excitatory spontaneous synaptic currents (mEPSCs) in neurons of adult (91% of cells tested), old (85% of neurons), and very old mice (77% of neurons). These residual non-glutamatergic mEPSCs were abolished by application of specific P2XRs antagonists PPADS (10 μM) and 5-BDBD (5 μM) in neurons of adult, old and very old mice (correspondingly 15,11 and 11 cells). Based on this results as well as our previous work (Pankratov et al., 2003, 2007; Lalo et al., 2014a, 2016), the spontaneous inward currents observed in the neocortical neurons in the presence of glutamatergic and GABAergic antagonists can be confidently attributed to the P2X receptors.

The spontaneous purinergic currents recorded in neurons of the adult SH mice under baseline conditions had an average amplitude of 8.1 ± 2.5 pA and an average decay time of 9.8 ± 2.9 ms (*n* = 12). We previously showed (Lalo et al., 2014a) that in neurons of young mice spontaneous purinergic currents could be divided into two populations: events of larger amplitude and faster decay kinetics and events of smaller amplitude and slower decay kinetics; the former population comprised events of synaptic origin whereas the latter population was elicited by exocytosis of ATP from astrocytes. Existence of two population of events manifested in the appearance of two peaks at amplitude and decay time distribution histograms (Lalo et al., 2014a). Activation of Ca^2+^-signaling in astrocytes, in particular via astroglia-specific PAR-1 receptors, dramatically increase the frequency of the smaller-and-slower currents, verifying their astroglial origin (Lalo et al., 2014a).

In consistence with our previous findings in young mice (Lalo et al., 2014a), the amplitude distribution of purinergic currents in pyramidal neurons of adult animals (Figure 4A1) exhibited a bimodal pattern with minor peak of smaller amplitude (5.5 ± 1.6 pA) and major peak of larger amplitude (8.7 ± 2.3 pA, *n* = 12). The distribution of mEPSCs decay time in these neurones exhibited peaks at 9.1 ± 1.4 ms and 21.8 ± 5.7 ms. The number of purinergic events of smaller amplitude and slow kinetics dramatically increased upon activation of astrocytes with PAR-1 agonist TFLLR (10 μM) which manifested in the enhancement of the corresponding peaks in the amplitude and decay time distributions (Figure 4B1). Hence, both in young and old mice (Figure 4A2–B3), quantal purinergic currents originating from the release of ATP from synaptic terminals and astrocytes can be distinguished by their biophysical properties. The baseline spontaneous purinergic currents of faster kinetics and larger amplitudes represent events elicited by release of ATP from synaptic terminals (referred further as purinergic mEPSCs) whereas the currents of slower kinetics, whose frequency increased upon stimulation of astrocytes with TFLLR, originate from glial release of ATP (referred further as glia-induced currents, GICs).

Thus, we used the amplitude and frequency of the baseline mEPSCs as post- and presynaptic readouts for ATP-mediated synaptic transmission (Figure 4C). Conversely, the TFFLR-induced elevation in the GICs frequency was used as a readout for astrocytic ATP release (Figure 4D). The mean amplitude and frequency of purinergic mEPSCs underwent significant decrease in the neurons of old and very old SH mice (Figure 4A2,B2,C); this result suggests that aging can affect both the surface density of synaptic P2X receptors and probability of ATP release from synaptic terminals. In parallel, the frequency of TFLLR-induced purinergic GICs exhibited strong age-dependent decrease (Figure 4A2,B2,D). This results implies that glia release of ATP can undergo substantial decline with aging which closely agrees with our previous data obtained using ATP microelectrode biosensors (Lalo et al., 2018).

Exposure to EE and CR caused an enhancement in the purinergic mEPSCs and GICs in all age groups, especially in the old and very old mice (Figure 4A3,C,D). In line with their effects on functional expression of P2X receptors in astrocytes (Figure 1), EE and CR substantially increased the amplitude of neuronal mEPSCs (Figure 4C). However, the effect of EE and, especially CR, on synaptic and astroglial release of ATP were not that prominent. Both treatments did not have significant effect on the frequency of mEPSCs (Figure 4C) and just moderately enhanced the frequency GICs in old and very old mice (Figure 4D). The weaker effects of EE and CR on the synaptic and astroglial exocytosis of ATP in the old age might be attributed to the general decline in metabolic functions of aging brain (Mercken et al., 2012; Lopez-Otin et al., 2016) which would very likely affect the production of ATP and its accumulation in synaptic/glial vesicles.

Combined together, our results demonstrate that the purinergic component of synaptic transmission and astrocyte-to-neuron communications can undergo substantial decline in the aging brain. It was shown previously in young mice that activation of post-synaptic purinoreceptors can up-regulate AMPA-receptor-mediated synaptic currents and down-regulate GABAergic inhibition (Boue-Grabot and Pankratov, 2017). Hence, one might expect astrocyte-driven purinergic modulation to change in the adult and very old mice.

### Changes in the Purinergic Modulation of AMPA Receptor-Mediated Synaptic Currents

**FIGURE 5:**
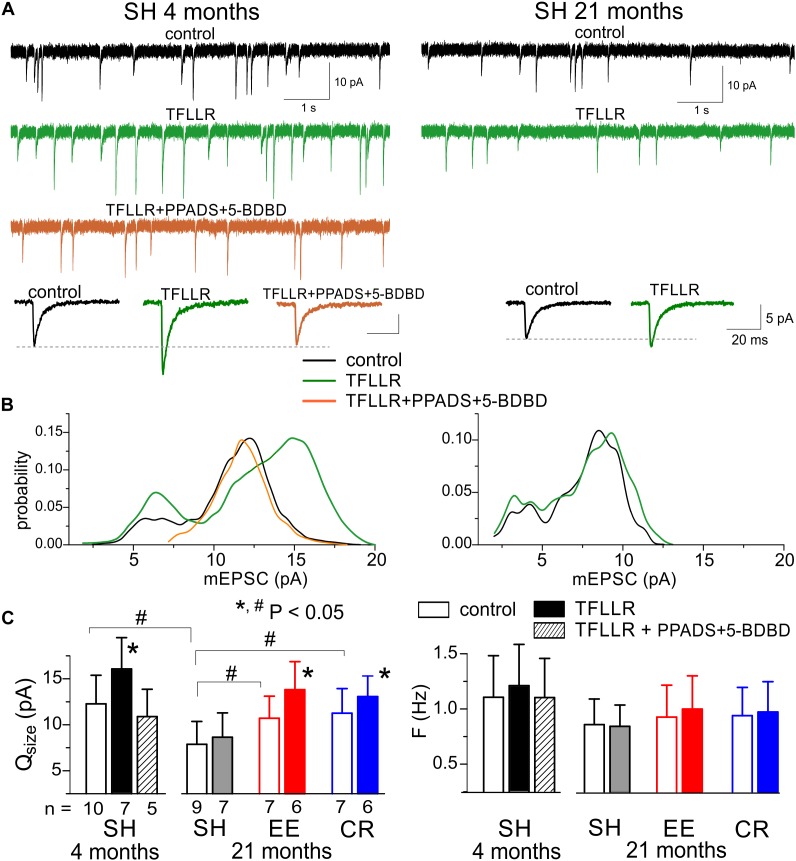
Astrocyte-driven modulation of excitatory synaptic currents in neocortical neurons. The AMPA receptor-mediated spontaneous mEPSCs were recorded in the neocortical neurons of adult (4 months) and very old (21 months) mice at –80 mV in presence of 100 μM picrotoxin, 1 μM TTX, and 30 μM D-APV. Astroglial modulation of mEPSCs was triggered by 3 min-long bath application of TFLLR. **(A)** the representative whole-cell currents recorded in the pyramidal neurons before (baseline) and 1 min after application of TFLLR alone or in the presence of P2X receptor antagonists PPADS and 5-BDBD. The insets below show the average mEPSCs waveforms recorded under different conditions. **(B)** The corresponding amplitude distributions (probability density functions calculated for 100–300 events registered over 5 min period). Note the significant increase in the mEPSCs amplitude after application of TFLLR in the neurons of adult mice and the lack of changes when TFLLR was applied in the presence of P2X receptor blockers. The post-synaptic alteration in the quantal size of mEPSCs is evidenced by rightward shift of the peak of amplitude distribution. **(C)** Diagrams show the quantal size and frequency of AMPAR-mediated mEPSCs in adult and very old mice of different environment groups; the data are shown as mean ± SD for the number of neurons indicated below. The hush symbols (#) indicate statistical significance (*P* < 0.05) of the difference between the age and environment groups. Asterisks (^∗^) correspondingly indicate statistical significance (*P* < 0.05) of the effect of TFLLR (as compared to the control).

We recorded the AMPA receptor-mediated miniature spontaneous synaptic currents in the pyramidal neurons at membrane potential of -80 mV in presence of picrotoxin and TTX. Under this conditions, the main fraction of spontaneous events was represented by the AMPA- receptor-mediated mEPSCs, which could be easily discriminated from the purinergic events by much larger quantal size, as evidenced by Figure 4A1,B1. In the adult mice, the mean amplitude of AMPAR-mediated mEPSCs showed significant increase (up to 25–30%) upon activation of astrocytes with TFLLR (Figure 5A,C); the mean mEPSCs frequency did not undergo marked changes. Analysis of the mEPSCs amplitude distribution showed the considerable rightward shift after TFLLR application indicating the increase in the quantal size. The effect of TFLLR was abolished by inhibition of P2X receptors with PPADS and 5-BDBD (Figure 5C). These results verify the post-synaptic locus and purinergic mechanism of the astroglia-driven enhancement of excitatory synapses.

Importantly, the TFLLR-induced modulation of AMPAR-mediated mEPSCs was significantly decreased (Figure 5A,C) in the very old mice where neuronal purinergic signaling and exocytosis of ATP from astrocytes is impaired (Figure 4). One might expect that manipulations which ameliorate age-related decline in purinergic signaling would also rescue purinergic modulation in the old mice. Indeed, exposure to EE significantly increased the effect of TFLLR on the quantal size of glutamatergic mEPSCs. The CR had much less prominent but still statistically significant effect (Figure 5C).

### Changes in the Purinergic Modulation of GABAergic Inhibition

**FIGURE 6:**
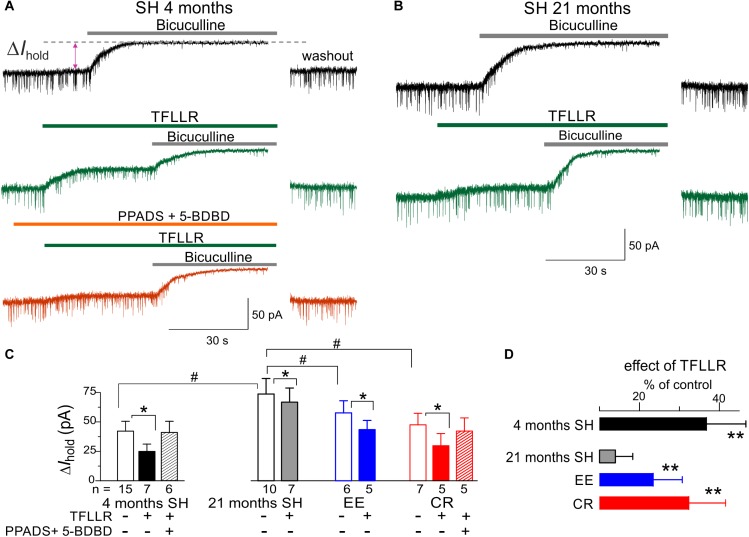
Astrocyte-driven modulation of tonic GABAergic currents in neocortical neurons. **(A,B)** The tonic GABA-mediated currents were evaluated by upward shift in the whole-cell holding current (ΔI_hold_) caused by application of bicuculline (50 μM) to the layer 2/3 pyramidal neurons in brain slices of adult (4 months) and very old (21 months) mice. Bicuculline was applied either in control (upper traces) or after activation of astrocytic signaling by 10 μM TFLLR (middle traces, *green*) or after application of TFLLR in presence of P2X receptors antagonist PPADS and 5-BDBD (bottom trace, *orange*). Currents were recorded in presence of NBQX (50 μM) and D-APV (30 μM) at holding potential of –80 mV. **(C)** The average amplitude of tonic GABA-mediated current measured in the mice of different age and environment groups; the data are shown as mean ± SD for the number of neurons indicated below. The hush symbols (#) indicate statistical significance (*P* < 0.05) of the difference between the age and environment groups. Asterisks (^∗^) correspondingly indicate statistical significance (*P* < 0.05) of the effect of TFLLR (as compared to the control). **(D)** The pooled data (mean ± SD) on the relative effect of TFLLR on the tonic currents. Asterisks (^∗∗^) indicate the statistical significance (*P* < 0.01) of difference from the very old mice of standard housing (21 months SH). Note that in the very old SH mice, the tonic inhibitory current was significantly up-regulated and activation of astroglial Ca^2+^ had much smaller effect on the tonic current than in the adult mice. The impact of aging on the TFLLR-induced modulation of the tonic current was ameliorated in the EE and CR mice.

As a readout for astroglia-driven modulation of GABAergic inhibition, we evaluated the TFLLR-induced modulation of tonic bicuculline-sensitive inhibitory currents, using experimental paradigm similar to our previous work in the young mice (Lalo et al., 2014a). Activation of astroglial PAR-1 receptors by TFLLR, which has been shown to induce ATP release (Lalo et al., 2014a), caused a significant decrease in the tonic inhibitory currents in the neocortical pyramidal neurons of adult mice (Figure 6A). This effect was occluded after inhibition of P2 purinoreceptors with PPADS and 5-BDBD, confirming the crucial role astrocyte-derived ATP (Figure 6A,C).

We observed the substantial up-regulation in the baseline tonic inhibitory currents in the neurons of very old mice which was accompanied by the marked attenuation of the effect of TFLLR (Figure 6B). These alterations resembled the previously reported phenotype (Lalo et al., 2014a) of mice with the deficit of astrocytic exocytosis of ATP (dnSNARE line). In line with data on modulation of excitatory currents, the astroglia-driven modulation of tonic inhibition in the very old mice was rescued by EE and CR (Figure 6C,D). In contrast to experience-dependent changes in the excitatory currents (Figure 5), the effects of CR on the baseline tonic inhibition and its TFLLR-induced attenuation were much stronger than effects of EE (Figure 6D).

## Discussion

Taken together, our results show that purinergic component of bi-directional communications between astrocytes and neurons can undergo marked remodeling across a lifetime and, in particular, in response to environment and experience. The data on age-dependent alterations in the P2X and P2Y receptors-mediated evoked and spontaneous signaling in astrocytes presented above (Figure 1, 2) closely agree with our previous data on alterations in the ionotropic astrocytic signaling (Lalo et al., 2011a). We observed the general trend of significant decrease in the astrocytic purinergic signaling in the old and very old age which is in line with other reports on detrimental morphological and molecular changes in the astrocytes (Lalo et al., 2011a, 2014b; Rodriguez et al., 2014; Verkhratsky et al., 2014). These data suggest that ability of astrocytes to monitor and integrate activity of neighboring neuronal networks, which is crucial for many astroglia functions, can be compromised in the aged brain. So, the age-related decline in the astroglial purinergic signaling could play a specific role in onset and progression of neurodegenerative disorders.

Our results (Figure 1, 3) also suggest that purinergic P2X and P2Y receptors can bring considerable contribution into Ca^2+^-signaling in cortical astrocytes across a lifetime. We would like to note that, while the role for P2Y receptors in glial signaling is widely recognized (Abbracchio et al., 2009; Butt, 2011; Araque et al., 2014; Verkhratsky et al., 2017), a contribution of ionotropic P2X receptors remains often overlooked. This may result from some confusion about the specificity of the most frequently used and highly efficient P2Y1 antagonist MRS2179 which was applied in many cases in the overkill concentrations (>1 μM). At such concentrations, MRS2179 can also inhibit the P2X1 subunit-containing receptors (Brown et al., 2000), which accordingly to our results, mediate the substantial part of ionotropic Ca^2+^-signaling in astrocytes (Lalo et al., 2008; see also Figure 1, 3). So, usage of MRS2179 at high concentrations can lead to overestimation the P2Y-mediated component for the expense of P2X-mediated component. To circumvent this problem, we used the more specific P2Y1 antagonist MRS2279 (Boyer et al., 2002). Our present results (Figure 1, 3) confirm our previous conclusions on the importance of P2X receptor-mediated Ca^2+^-influx in the function of neocortical astrocytes across a lifetime (Lalo et al., 2011a). Interestingly, the ionotropic component of purinergic Ca^2+^-signaling showed sharp age-related changes (as compared to P2Y-mediated component) and turned out to be highly responsive to the beneficial effects of EE and CR. This may imply a novel physiological role for P2X receptors in astrocytes which are yet to be investigated.

Rather surprisingly, our data do not show any significant contribution of P2X7 receptors into spontaneous or evoked purinergic signaling in neocortical astrocytes (Figure 2) that may seem to disagree with numerous reports of P2X7 expression in glial cells (Butt, 2011; Rivera et al., 2016). Our results also support the previously expressed cautions against usage of BzATP as a definitive proof of P2X7-mediated activity (Anderson and Nedergaard, 2006). We would like to emphasize that present work has been focused primarily on changes in the purinergic signaling during physiological aging; particularly we optimized our tissue preparation procedure to minimize the risk of inflammation and ischemia in the brain slices of old mice. Accordingly to our estimations (Lalo et al., 2014a), the concentration of ATP in the extracellular space after vesicular release from neurons or astrocytes cannot exceed 10–100 μM which is not sufficient to activate the P2X7 receptors. The P2X7-mediated signaling, however, is usually associated with various pathological conditions such as neuro-inflammation, epilepsy and ischemia, where excessive ATP release can occur (Abbracchio et al., 2009; Rivera et al., 2016). The plausible function of astroglial P2X7 receptors upon these conditions surely deserves a further study, especially in the context of brain aging.

Our observation of decrease in astroglia-driven P2X-mediated currents in pyramidal neurons goes in line with data on the decline in astroglial exocytosis of ATP and other gliotransmitters (Lalo et al., 2014b, 2018). In parallel, we observed a decrease in the quantal size of neuronal purinergic currents, which was, most likely, due to decrease in the density of post-synaptic P2X receptors. Hence, all parts of purinergic signaling with “tripartite synapse” can undergo significant decline with aging. Importantly, the purinergic signaling within a “tripartite synapse” turned out to be very responsive to EE and CR both at the post-synaptic and astroglial side (Figure 1, 4).

**FIGURE 7:**
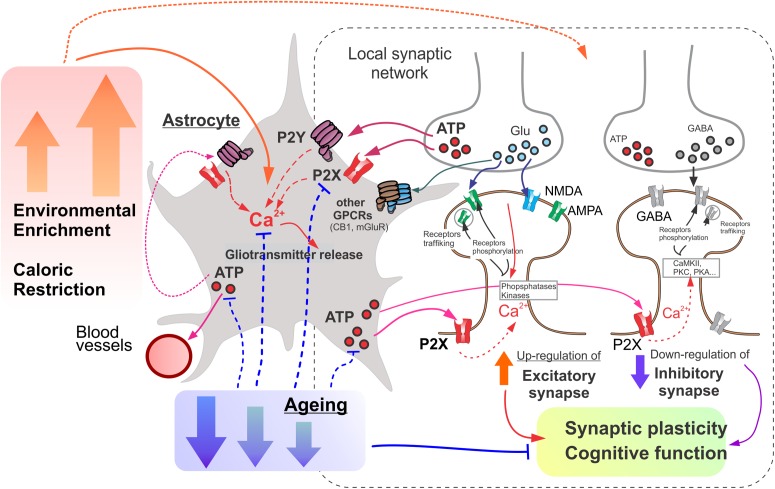
The putative mechanisms underlying the involvement of astroglial purinergic signaling on the age- and experience-related brain plasticity. Astrocytes act a “Brain Hub” receiving signals from neurons and regulating synaptic transmission, neuronal metabolism, and neurovascular coupling. Astroglial purinergic Ca^2+^ signaling is activated by ATP released from synaptic terminals as a co-transmitter and ATP released from astrocytes. Astrocytes up-regulate excitatory and down-regulate inhibitory synapse by releasing ATP (as well as other gliotransmitters) thereby facilitating synaptic plasticity and cognitive functions. Age-related attenuation in astrocytic purinergic signaling compromises glial control of synaptic homeostasis. Environmental enrichment (EE) and caloric restriction (CR) enhance purinergic signaling in astrocytes and ameliorate the age-related decline in their function.

Although (patho)physiological consequences of alterations of purinergic glia-neuron communications are yet to be fully understood, our data can give few important insights into this topic. In particular, our data show that ATP, in particular glia-derived, does not cease to play a neuro-modulatory functions in the synapses of aging brain but extent of purinergic modulation can decrease due to decline in the release of ATP and density of post-synaptic ATP receptors (Figure 5). Our present (Figure 4) and previous data (Lalo et al., 2014b, 2018) demonstrate that release of ATP can undergo significant decline with aging. This in turn can cause an impairment of purinergic astroglia-driven modulation of synaptic signaling, manifested in particular in the decrease in the glutamatergic (Figure 5, 7) and increase in the GABAergic signaling (Figure 6, 7). Such shift of the balance toward inhibition can affect the induction of long-term synaptic plasticity. So, the deficit in the purinergic component of glia-neuron communications could contribute to the age-related impairment of synaptic plasticity (Figure 7). This notion is supported by our previous data on the impairment of LTP in the neocortex of old mice which was rescued by additional stimulation of astrocytes or supplementing the ATP by exogenous non-hydrolysable analogs (Lalo et al., 2014b, 2018). This notion is further supported by our observations that manipulations facilitating neuronal P2X-mediated signaling and astroglial release of ATP, e.g., EE and CR, reversed the changes in excitatory and inhibitory synaptic signaling back to “younger” state (Figure 5, 6). Interestingly, that effect of CR on the GABAergic currents in the very old mice was unexcitingly high (being large than effect of EE) taking into account rather moderate effect of CR on neuronal P2X-mediated currents (Figure 4). This suggest than mechanisms of beneficial effects of CR on neuronal signaling might involve other molecular cascades, particular modulation of autophagic processes in astrocytes and neurons (Lopez-Otin et al., 2016; Nikoletopoulou and Tavernarakis, 2018).

## Conclusion

To conclude, our results strongly support physiological importance of astroglial purinergic signaling and glia-derived ATP for communication between astrocytes and neurons across a lifetime. The age-related decline in the purinergic component of glia-neuron interactions can lead to a dysregulation of excitatory and inhibitory synaptic signaling and thereby can be an aggravating factor, if not the cause, of cognitive decline in aging and neurodegenerative diseases. On another hand, our data demonstrate that EE and CR can enhance purinergic glia-neuron communications and ameliorate negative effects of aging on synaptic transmission.

## Data Availability

The datasets generated for this study are available on request to the corresponding author.

## Ethics Statement

All animal work was carried out in accordance with United Kingdom legislation and “3R” strategy; research did not involve non-human primates. This project was approved by the University of Warwick Animal Welfare and Ethical Review Body (AWERB), approval number G13-19, and regulated under the auspices of the United Kingdom Home Office Animals (Scientific Procedures) Act licenses P1D8E11D6 and I3EBF4DB9.

## Author Contributions

YP and UL conceived the study and wrote the manuscript. UL, AB, and YP performed the experiments and data analysis.

## Conflict of Interest Statement

The authors declare that the research was conducted in the absence of any commercial or financial relationships that could be construed as a potential conflict of interest.
